# Observational Studies and a Statistical Early Warning of Surface Ozone Pollution in Tangshan, the Largest Heavy Industry City of North China

**DOI:** 10.3390/ijerph10031048

**Published:** 2013-03-13

**Authors:** Pei Li, Jinyuan Xin, Xiaoping Bai, Yuesi Wang, Shigong Wang, Shixi Liu, Xiaoxin Feng

**Affiliations:** 1 College of Atmospheric Science, Lanzhou University, Lanzhou, Gansu 730000, China; E-Mails: lipei@dq.cern.ac.cn (P.L.); baixp11@lzu.edu.cn (X.B.); wangsg@lzu.edu.cn (S.W.); 2 State Key Laboratory of Atmospheric Boundary Layer Physics and Atmospheric Chemistry, Institute of Atmospheric Physics, Chinese Academy of Sciences, Beijing 100029, China; E-Mails: wys@dq.cern.ac.cn (Y.W.); lsx@dq.cern.ac.cn (S.L.); 3 Unit 93534 of PLA, Beijing 101212, China; 4 College of Material Science, Hebei United University, Tangshan, Hebei 063009, China; E-Mail: fxxipac@163.com

**Keywords:** Tangshan, ozone pollution, O_3_, nitrogen oxides, early warning, heavy industry city

## Abstract

Continuous measurements of surface ozone (O_3_) and nitrogen oxides (NO_X_) at an urban site (39°37′N, 118°09′E) in Tangshan, the largest heavy industry city of North China during summertime from 2008 to 2011 are presented. The pollution of O_3_ was serious in the city. The daily maximum 1 h means (O_3_1-hr max_) reached 157 ± 55, 161 ± 54, 120 ± 50, and 178 ± 75 μg/m^3^ corresponding to an excess over the standard rates of 21%, 27%, 10%, and 40% in 2008–2011, respectively. The total oxidant level (O_X_ = O_3_ + NO_2_) was high, with seasonal average concentrations up to 100 μg/m^3^ in summer. The level of O_X_ at a given location was made up of NO_X_-independent and NO_X_-dependent contributions. The independent part can be considered as a regional contribution and was about 100 μg/m^3^ in Tangshan. Statistical early warning analysis revealed that the O_3 _levels would exceed the standard rate by 50% on the day following a day when the daily average ozone concentration (O_3_mean_) exceeded 87 μg/m^3^ and the daily maximum temperature (T__max_) exceeded 29 °C. The exceed-standard rate would reach 80% when O_3_mean_ and T__max_ exceeded 113 μg/m^3^ and 31 °C. Similarly, the exceed-standard rate would reach 100% when O_3_mean_ and T__max_ exceeded 127 μg/m^3^ and 33 °C, respectively.

## 1. Introduction

Ozone (O_3_) in the lower part of the atmosphere (troposphere) is one of the most widespread global air pollution problems today. Evidence for the adverse effects of O_3_ on both human health and the environment at existing concentrations can currently be found in many developed countries, as well as developing countries [1,2,3]. Therefore, many countries and regions have established appropriate environmental standards for this pollutant, but human exposure to high concentrations of ground level ozone continues to be a serious problem in many areas in the US and China, despite the implementation of government-mandated emission control strategies [4,5]. The control of ground level ozone is more difficult than for many other primary pollutants because ozone is a secondary pollutant. The formation of ground level ozone depends on the intensity of solar radiation, the absolute concentrations of its precursors such as nitrogen oxides (NO_X_) and volatile organic compounds (VOCs), and the VOCs/NO_X_ ratios [6,7,8]. Owing to the chemical coupling of O_3_, nitric oxide (NO), and nitrogen dioxide (NO_2_), the levels of O_3_ and NO_2_ are inextricably linked. Therefore, the response to reductions in the nitrogen oxides emissions is remarkably not linear, and any resultant reduction in the level of nitrogen dioxide is invariably accompanied by an increase in the air concentration of ozone [9,10]. Moreover, the increasing O_3_ background concentration influences local levels of O_3_, and NO_2_ and the efficiency of local emission controls. It is therefore necessary to obtain a thorough understanding of the relationships and the chemical coupling among O_3_, NO, and NO_2_ under various atmospheric conditions. 

A number of previous studies showed that tropospheric O_3_ was increasing in many countries and regions [9,10,11,12,13,14]. Recently, many Chinese scientific researchers have monitored and analyzed the pollution of O_3_ in the northwest, southeast and Beijing-Tianjin-Hebei regions of China [11,13,15,16,17,18,19]. Some studies found that the mountainous northwest area of the Beijing-Tianjin-Hebei region was a storage area for O_3_, where O_3_ and O_X_ (=O_3_ + NO_2_) levels were remarkably higher than in the Beijing-Tianjin-Hebei plain [15,18], and O_3_ has become a serious threat to the environment during summertime in certain cities of China. The Beijing-Tianjin-Hebei Atmospheric Environment Monitoring Network was established by the Institute of Atmospheric Physics, Chinese Academy of Sciences (IPA, CAS). The results of the network showed that the complex atmospheric pollution exhibited high concentrations of O_3_ and fine particles and oxidation in summer, with ubiquitous regional sources [18]. To achieve a reduction in the comprehensive pollution of O_3_ and particulate matter will be a very serious and difficult challenge in the region [16,18,19].

Tangshan is the largest heavy-industry city with a long history in the Beijing-Tianjin-Hebei regions. Coal emissions and photochemical smog pollution have increased dramatically with the rapid growth of Tangshan’s industrial sector since the 1990s. According to the 2010 Report of the Tangshan Environmental Protection Agency (TEPA), the ambient air quality of Tangshan city has improved gradually in recent years [20]. However, the environmental pollution caused by industrial and transport emissions should not be viewed optimistically. The 2016 International Horticultural Exposition will soon be hosted by Tangshan City, and there is a great need to assess and improve the air quality of the city. Based on the monitoring data from 2008 to 2011, the present study investigated the relationships between ambient levels of O_3_, NO, NO_2_, and O_X_, and developed an early warning assessment method to provide a scientific basis for the prevention of air pollution in Tangshan. 

## 2. Materials and Methods

**Figure 1:**
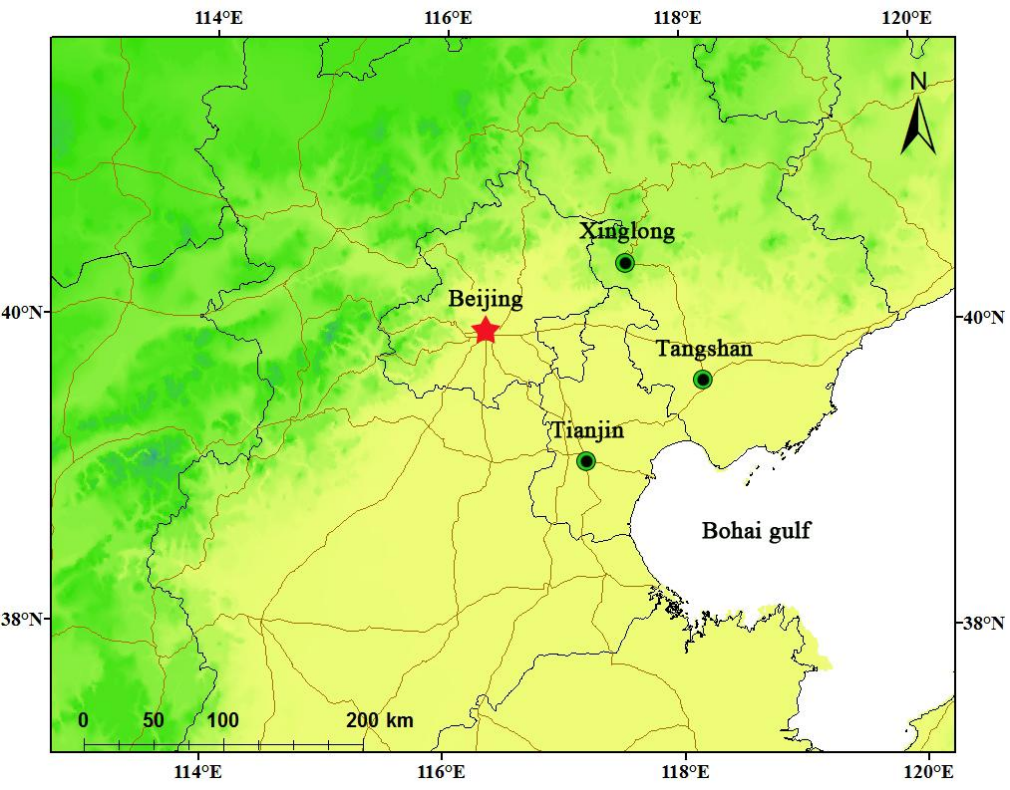
Location of Tangshan station in the Beijing-Tianjin-Hebei region.

The data were collected from 2008 to 2011 at the Tangshan station, a site of the Air Quality Monitoring Network, which was established by IPA, CAS and Hebei United University. Figure 1 shows the location of the Tangshan station within the Beijing-Tianjin-Hebei region. 

The station was in the urban center, located on the top of the four-layer office building of Hebei United University (39°37′N, 118°09′E, the air intake was 12 m from the ground). Within a radius of about 5 km of the measured area, the land was relatively flat with some low residential buildings and commercial buildings, without remarkable point emission sources. The station was equipped with the online robotic instruments from Thermo Scientific (Franklin, MA, USA), including a 49I UV photometric O_3_ Analyzer and 42I NO-NO_2_-NO_X_ precision chemiluminescence analyzers. Quality control checks, including automatic zero calibration and span checks of gas analyzers, were performed daily, and manual calibrations with standard gases were conducted weekly. Multi-point calibrations of the O_3_ analyzer used an O_3_ calibrator (TEI Model 49CPS, Franklin, MA, USA). The NO_X_ analyzers have been zero-checked and span-checked using a zero gas generator (TEI Model 111, Franklin, MA, USA) and an internal O_3_ source of a multi-gas calibrator (TEI Model 146C, Franklin, MA, USA) with NO standard (National Centre for Standard Materials, Beijing, China). Sampling methods and instrument protocols, as well as quality assurance/quality control (QA/QC) procedures for air quality monitoring, were be executed based on the Chinese National Environmental Protection Standard, Automated Methods for Ambient Air Quality Monitoring. The real-time data was collected and transferred via the internet. The meteorological data were from actual Meteorological Information Comprehensive Analysis and Process System (MICAPS), which was established by the National Meteorological Center of China. 

## 3. Results and Discussion

### 3.1. Variation of O_3_ in Tangshan during the Summertime

Table 1 shows the mean concentrations of atmospheric pollutants observed in Tangshan during the observation period. Total oxidant was taken to be the sum of O_3_ and NO_2_. The daily average (O_3_mean_), daily maximum 1 h mean (O_3_1-h max_) and daily maximum 8 h mean (O_3_8-h max_) were used to describe the changes of O_3_ concentration. The seasonal average concentrations of O_3_mean_, O_3_1-h max_, and O_3_8-h max_ were 69 ± 28, 154 ± 61, and 124 ± 51 μg/m^3^ in summer during the 4 years study period, respectively. The lowest value of O_3_mean_ was 53 ± 22 μg/m^3^ in 2010, and the highest was 79 ± 35 μg/m^3^ in 2011. The result is consistent with a previous study [17]. Xin *et al.* found that the daily average concentration of O_3_ were 69 ± 22 μg/m^3^ in Beijing, 73 ± 16 μg/m^3^ in its surrounding area (including the Tangshan city), and 100 ± 25 μg/m^3^ in Xinglong during the Beijing 2008 Olympic Games, respectively [18]. Tang *et al.* found that were 66 μg/m^3^ and 54 μg/m^3^ in Beijing and its surrounding area from July to September during 2001–2006, respectively [16,19]. Compared with the above results, the pollution of O_3_ was very serious in the city, the same as in Beijing and its surrounding area.

**Table 1:** The mean concentrations of atmospheric pollutants observed in Tangshan in summer (μg/m^3^).

Period	O_3_			NO	NO_2_	NO_X_	O_X_
	O_3_mean_	O_3_1-h max_	O_3_8-h max_				
2008: 06/01–09/30	75 ± 25	157 ± 55	129 ± 46	5 ± 4	41 ± 10	46 ± 13	116 ± 27
2009: 07/13–09/30	69 ± 29	161 ± 54	126 ± 52	7 ± 5	43 ± 10	50 ± 13	113 ± 28
2010: 06/01–09/30	53 ± 22	120 ± 50	97 ± 42	6 ± 5	47 ± 13	54 ± 14	100 ± 32
2011: 06/01–08/10	79 ± 35	178 ± 75	143 ± 64	4 ± 4	39 ± 10	44 ± 12	118 ± 36
Mean	69 ± 28	154 ± 61	124 ± 51	5 ± 5	43 ± 11	49 ± 13	112 ± 31

**Figure 2:**
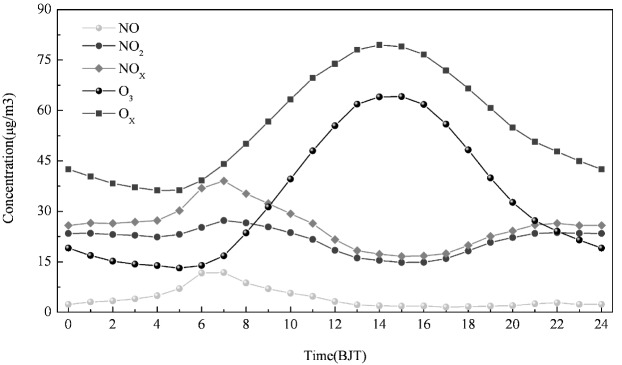
Daily variation of mean concentrations of O_3_, NO, NO_2_, NO_X_, and O_X _during the summertime in Tangshan.

The hourly averaged concentrations of O_3_, NO, NO_2_, NO_X_, and O_X_ are shown in Figure 2. A distinct daily cycle of the pollutants was observed. In general, the daily cycle of O_3_ reached a peak during the middle of the day and had lower nighttime concentrations. The O_3_ concentration slowly increased as the sun rose, reached the maximum at 14:00, and then slowly decreased until 05:00 of the next morning. The daily cycle of NO, NO_2_, and NO_X_ concentration showed morning peaks and evening peaks. It can be seen that nitrogen oxides increased with increasing traffic in the morning and evening. NO is converted to NO_2_ via the reaction with O_3_ and during daylight hours, and NO_2_ is converted back to NO by photolysis, which also regenerates O_3 _ [6,7,21,22]. As long as O_3_ was present in excess, NO did not rise throughout the day. Since the photolysis rate at nighttime is zero there is net removal of O_3_ by NO. The daily variation patterns of O_3_ and O_X_ during the study period were similar.

### 3.2. Chemical Coupling of O_3_, NO and NO_2_

Photochemical oxidants play key roles in the atmospheric pollution over urban areas. Among these, O_3_ and NO_2_ are important. Produced in the atmosphere through a set of complex reactions [9,10], they are capable of causing adverse impacts on human health and the environment. It is well established that the inter-conversion of O_3_, NO and NO_2_ under atmospheric conditions is generally dominated by the following Equations [21,22]:

NO + O_3_ → NO_2_ + O_2_ (1)

NO_2_ + hγ (+O_3_) → NO + O_3_ (2)

In Equations (1)–(2), NO is initially oxidized by O_3_ to form NO_2_ which is then further converted to O_3_ through photolysis. Moreover, O_3_ can be consumed by a set of photochemical reactions. In polluted regions, however, peroxy radicals (RO_2_) participate in Reaction (1) instead of O_3_ (NO + RO_2_ → NO_2_ + RO). Under these conditions, pollution is caused by the accumulation of O_3_, if the concentration of O_3_ has reached a certain level. An analysis of the NO_X_ cycle in the atmosphere can contribute to understanding the process of O_3_ pollution in Tangshan. The previous Equations cycle (1)–(2) allows a determination of the concentrations of these chemical species in this photostationary state, as shown by the following expression [19,21]:

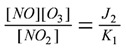
(3)

**Figure 3:**
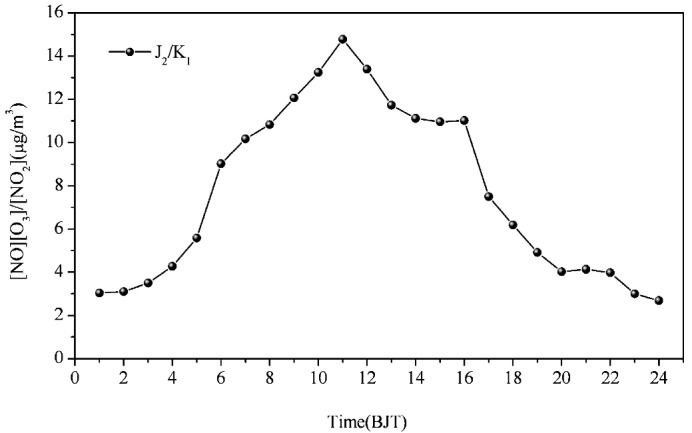
Daily variation of mean values of J_2_/K_1_ (μg/m^3^).

In this expression, J_2_ is the rate of NO_2_ photolysis and K_1_ the rate coefficient for the reaction of NO with O_3_. Coefficient J_2_ is a function of the solar radiation intensity. Coefficient K_1_ is a function of the temperature. The variation of the mean values J_2_/K_1_ is shown in Figure 3. The range of the mean J_2_/K_1_ was from 3 to 15 μg/m^3^ and the maximum occurred at 11:00. 

**Figure 4:**
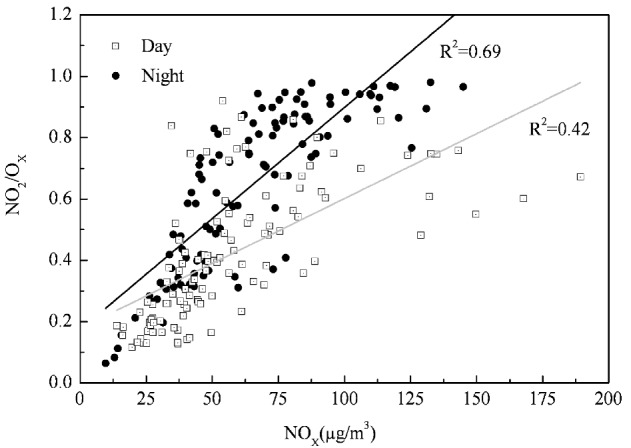
Variation of [NO_2_]/[O_X_] as a function of NO_X_.

On the basis of the photostationary state relationship, it is possible to infer an expected variation of daily average [NO_2_]/[O_X_] values with [NO_X_]. The variation of [NO_2_]/[O_X_] with NO_X_ concentration is shown in Figure 4. It could be seen that the ratio of [NO_2_]/[O_X_] as a function of NO_X_ was higher at night than during day. The greater portion of O_X _was in the form of NO_2_ at night. The day and night variation could be explained in terms of variation of the photolysis rate constant (J_2_), which was a function of the solar radiation intensity and the time required for conversion of NO to NO_2_ (related to wind speed) [10]. At night O_3_ and NO cannot coexist and the conversion to NO_2_ occurs in a short time. Thus, more NO_X_ was speciated as NO_2_ at night. Further, this showed that the reaction with freshly emitted NO and O_3_ via the O_3_ channel mainly controlled NO_2_ concentration and the O_3_ remaining after reaction with NO determined O_3_ concentration. This implied that the contribution of the NO_X_ channel for O_X_ production was major and the radical channel was minor. The residual O_3_ remaining after the NO-NO_2_-O_3_ reaction controlled the O_3_ concentration in the urban atmosphere during the monitoring period. 

**Figure 5:**
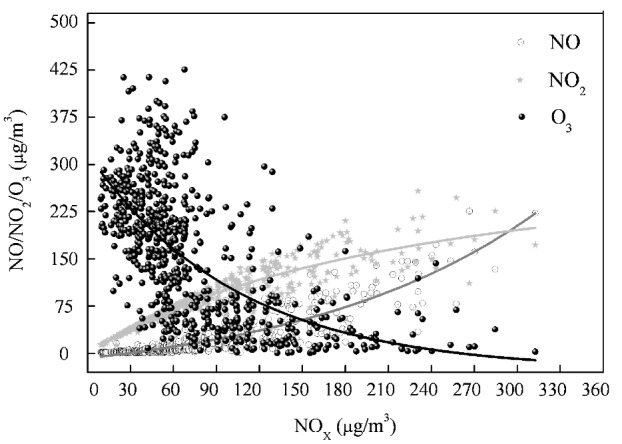
Variations of daylight average concentrations of O_3_, NO, and NO_2_ with the level of NO_X_.

The observed variation of daylight average concentrations of O_3_, NO, and NO_2_ with the total level of NO_X_ is shown in Figure 5. The lines in Figure 5 were fitted using the multiple regression method to investigate the assumption of a photostationary state. The curves of [O_3_] and [NO] indicated that the [NO_X_] crossover point occurred at about 160 μg/m^3^. When [NO_X_] < 160 μg/m^3^, O_3_ levels were higher than the NO levels, whereas NO dominated at higher [NO_X_]. The intersection point of the curves represented the two oxidants as [NO_X_] = 100 μg/m^3^. When [NO_X_] < 100 μg/m^3^ O_3_ was the dominant form and NO_2_ dominated at higher [NO_X_] levels. This pattern was consistent with other research results [10,23,24], although the intersection points vary with local conditions.

### 3.3. Local and Regional Contributions to Oxidant

**Figure 6:**
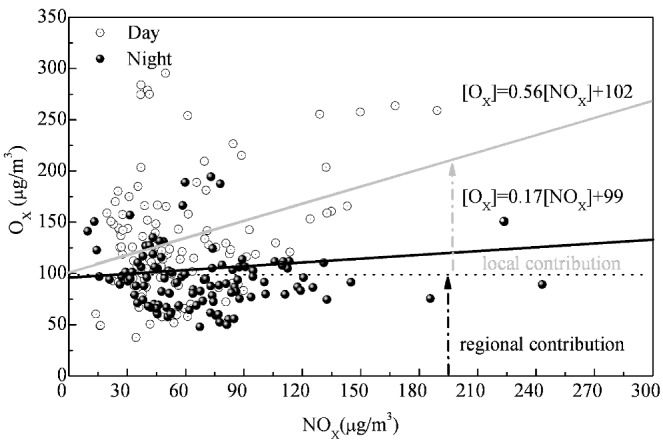
Variation of daily average O_X_ with level of NO_X_.

The variation of daylight and nighttime values of O_X_ concentration with the level of NO_X_ are included in Figure 6. 

Total [O_X_] increased with NO_X_, where the data were fitted by linear regression. Due to the influence of the photochemical reactions on the formation of O_3_, there were differences in the values and the linearly fitted equations between daytime and night. It could be observed that the total O_X_ at a given location had an NO_X_-independent contribution, and an NO_X_-dependent contribution. The former was effectively a regional contribution which equated to the regional background O_3_ level, whereas the latter was effectively a local contribution which was correlated with the level of primary pollution. It was found that the NO_X_-dependent local contribution to O_X_ at nighttime was 25% lower than during the daytime. However, the regional contribution, approximately to 100 μg/m^3^, was almost equivalent during both day and night. The regional contribution to O_3_ was highly consistent with the values observed at Xinglong, which is the background station of the Beijing-Tianjin-Hebei region [18]. This result implied that O_3_ pollution had a similar source throughout the Beijing-Tianjin-Hebei region. It implied that the air quality problem in Tangshan was not only a local problem mainly from street-level pollutions, but also a regional problem from the Beijing-Tianjin-Hebei region. The territorial character of O_3_ pollution was consistent with the results reported by Xin *et al.* [18].

### 3.4. Ozone Assessment and Early Warning

Based on the National Ambient Air Quality Standards (NAAQS) which will be implemented in 2016 in China, the urban air quality standards of level II for the 1 h average and 8 h average of the O_3_ concentration were used in this paper. These standards specify a concentration not to exceed 160 μg/m^3^ and 200 μg/m^3^, respectively. Table 2 shows the results for the O_3_ exceed-standard days and exceed-standard rates based on the level II standards. The exceed-standard rates were very high in summer and were 22%, 28%, 10%, and 41% during 2008, 2009, 2010, and 2011, respectively. The highest value (38%) appeared in June, and the lowest value (8.3%) appeared in September. Except in 2010, the number of exceed-standard days during the past 4 years exceeded 25, and the exceed-standard rates exceeded 20% (Table 2). The exceed-standard rates calculated by 8 h average standard were 2.2% higher than those by 1-hour average standard of level II. Therefore, the Tangshan government would need to assume stricter control of O_3_ pollution over the coming years.

**Table 2:** Exceed-standard days and exceed-standard rates in Tangshan during the past four years.

	2008		2009		2010		2011		Mean	
	No.	Rates	No.	Rates	No.	Rates	No.	Rates	No.	Rates
**1-h average**	25	21%	21	27%	10	10%	27	40%	83	23%
**8-h average**	29	24%	23	29%	10	10%	29	43%	91	25%
**Mean**	54	22%	44	28%	20	10%	56	41%	174	24%

Several methods were employed in ozone forecasting in many studies, such as Principal Components Analysis (PCA) which is a statistical technique used to investigate the structure of a data sets [25,26,27,28,29], Artificial Neural Networks (ANN) which is a mathematical model capable of determining a non-linear relationship between two data sets [30,31,32,33], Support Vector Machines (SVM) which have become more popular for air quality prediction [34,35,36], *etc.* Previous studies had shown that the temperature, humidity, and wind speed can affect the formation of O_3_, but high temperatures, low humidities and low wind speeds may not have contributed to a high concentration of O_3_ in all cases [7,37,38,39]. It was also indicated that the formation of O_3_ was a strongly coupled and complex nonlinear multivariable process [40]. 

We analyzed the meteorological factors selected to represent the relevant conditions, such as the temperature (T__mean_, T__max_), dew point temperature (Td__mean_), depression of the dew point (Ttd__mean_), wind speed (WS__mean_), visibility (Vis__mean_), 24 h isallobaric value (△P_24_), and 24 h isallotherm value (△T_24_). The O_X_mean_, O_3_mean_, NO__mean_, NO_2_mean_, NO_X_mean_, and [NO/NO_2_] _ _mean_ were also introduced in this paper. Table 3 shows the correlation coefficients between the selected factors on the current day and the O_3_ concentrations at the following day. 

**Table 3:** Coefficients of correlation between 15 factors and O_3_ concentrations in the next day.

	Factor	O_3_1-h__max_	O_3_8-h__max_		Factor	O_3_1-h__max_	O_3_8-h__max_
1	T__mean_	0.38 ^**^	0.39 ^**^	9	△T_24_	−0.01	−0.01
2	T__max_	0.45 ^**^	0.46 ^**^	10	O_X_mean_	0.45 ^**^	0.45 ^**^
3	Td__mean_	0.28 ^**^	0.24 ^**^	11	O_3_mean_	0.54 ^**^	0.57 ^**^
4	Ttd__mean_	0.11	0.13	12	NO__mean_	−0.24 ^**^	−0.24 ^**^
5	RH__mean_	−0.12 ^*^	−0.14 ^*^	13	NO_2_mean_	−0.19 ^*^	−0.22 ^*^
6	WS__mean_	−0.17 ^*^	−0.14 ^*^	14	NO_x_mean_	−0.27 ^*^	−0.27 ^*^
7	Vis__mean_	0.03	0.09	15	[NO/NO_2_]__mean_	−0.15 ^*^	−0.19 ^*^
8	△P_24_	0.08	0.09				

** Correlation is significant at the 0.01 level (2-tailed); * Correlation is significant at the 0.05 level (2-tailed).

**Figure 7:**
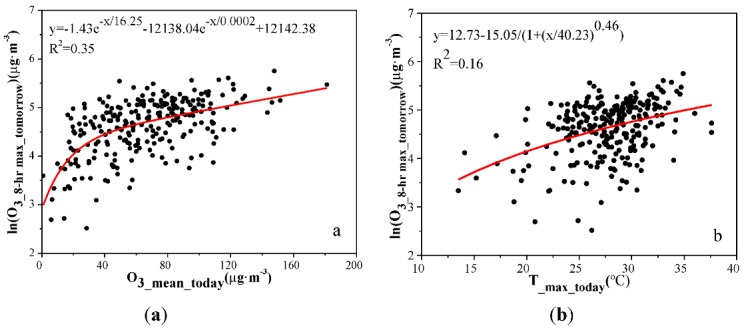
The changes of O_3_8-hr max_ at the following day in the response to (**a**) ozone concentration; (**b**) temperature on the first day during the study periods.

Figure 7 shows 2 two indicators which have been chosen to represent the O_3_ concentration on the following day. The maximum R-square value observed in these analyses 0.35, was found for O_3_mean_. The next highest value 0.15, was found for T__max_(Figure 7). For all of the functions used in the analysis, the remaining factors had consistently weak effects on the O_3_ concentration. It implied that O_3_mean_(X_1_) and T__max_(X_2_) had more significant effects on the O_3_ concentration of the next day. A multiple nonlinear regression equation corresponding to these factors was:

Y = 0.005X_1_^2^ + 0.487X_2_^2^ − 0.091X_1_X_2_ + 2.524X_1_ − 16.434X_2_ + 209.977 (R^2^ = 0.21, *P* < 0.001). (4)

Using the multiple nonlinear regression equation, we can estimate the exceed-standard rate of O_3_ in the next day. Under stable meteorological conditions, when the O_3_mean_ was less than 36 μg/m^3^ and T__max_ was less than 24 °C, the next day’s O_3_ concentration would not exceed the urban air quality standards of level II, the exceed-standard rate at the following day equivalent to 0. 

**Figure 8:**
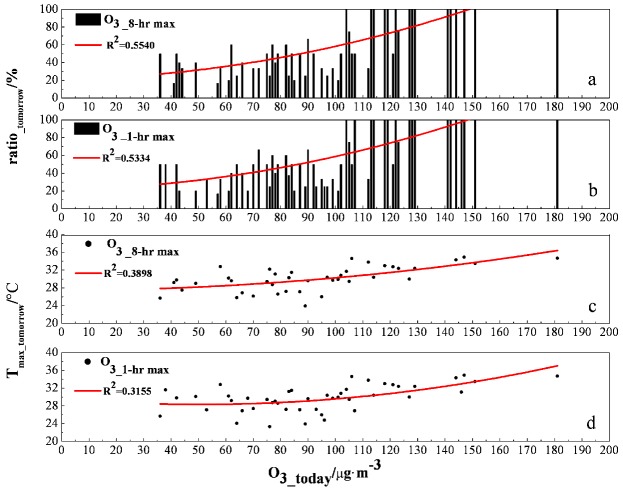
The corresponding exceed-standard ratios at the following day of (**a**) O_3___8-h max_ and (**b**) O_3___1-h max_ on the current day, and the corresponding max temperature on the current day when (**c**) O_3___8-h max_, and (**d**) O_3___1-h max_ exceed the standard at the following day.

Figure 8 shows the exceed-standard rates of O_3_ at the following day associated with an increase of 1 μg/m^3^ on the first day. The figure also indicated the changes of T__max_ as the concentration of O_3_ exceeded the standard. An exceed-standard rate of 50% can be defined as the level 1 early warning for O_3_ pollution, an exceed-standard rate of 80% can be defined as the level 2 early warning for O_3_ pollution, an exceed-standard rate of 100% can be defined as the level 3 early warning for O_3_ pollution. The following results were obtained from the curve fitting and statistical analysis used in this study. It showed that under stable meteorological conditions, if O_3_mean_ was less than 36 μg/m^3^ and T__max_ was less than 24 °C, the exceed-standard rate at the following day would be 0; if the O_3_mean_ was higher than 87 μg/m^3^ and T__max_ was higher than 29 °C, the exceed-standard rate would reach 50%; if the O_3_mean_ was greater than 113 μg/m^3^ and T__max_ was greater than 31 °C, the exceed-standard rate would reach 80%; if the O_3_mean_ was greater than 127 μg/m^3^ and T__max_ was greater than 33 °C, the exceed-standard rate would reach 100%. 

## 4. Conclusions

Tangshan city is the largest heavy-industry city in North China. In recent decades, the economical development was very rapid, leading to serious air pollution. The observations showed that the concentrations of O_3_ and O_X_ were very high in summer. The pollution of O_3_ in Tangshan was not only a local problem resulting mainly from street-level pollution, but also a regional problem from the Beijing-Tianjin-Hebei region. With the statistical analysis of the long-term data, we developed a simple method to assess the exceed-standard rate of O_3_ at the following day. The short-term early warning method would strengthen the capability to prevent regional atmospheric pollution accidents. In conjunction with other methods, the method could be used to estimate some governmental control strategies for photochemical pollutants in Tangshan city. 
